# Estimation of Left Ventricular End-Systolic Elastance From Brachial Pressure Waveform *via* Deep Learning

**DOI:** 10.3389/fbioe.2021.754003

**Published:** 2021-10-27

**Authors:** Vasiliki Bikia, Marija Lazaroska, Deborah Scherrer Ma, Méline Zhao, Georgios Rovas, Stamatia Pagoulatou, Nikolaos Stergiopulos

**Affiliations:** Laboratory of Hemodynamics and Cardiovascular Technology, Institute of Bioengineering, Swiss Federal Institute of Technology, Lausanne, Switzerland

**Keywords:** cardiac monitoring, convolution neural networks, cardiovascular modelling, non-invasive, contractility

## Abstract

Determination of left ventricular (LV) end-systolic elastance (E_*es*_) is of utmost importance for assessing the cardiac systolic function and hemodynamical state in humans. Yet, the clinical use of E_*es*_ is not established due to the invasive nature and high costs of the existing measuring techniques. The objective of this study is to introduce a method to assess cardiac contractility, using as a sole measurement an arterial blood pressure (BP) waveform. Particularly, we aim to provide evidence on the potential in using the morphology of the brachial BP waveform and its time derivative for predicting LV E_*es*_ *via* convolution neural networks (CNNs). The requirement of a broad training dataset is addressed by the use of an in silico dataset (*n* = 3,748) which is generated by a validated one-dimensional mathematical model of the cardiovasculature. We evaluated two CNN configurations: 1) a one-channel CNN (CNN_1_) with only the raw brachial BP signal as an input, and 2) a two-channel CNN (CNN_2_) using as inputs both the brachial BP wave and its time derivative. Accurate predictions were yielded using both CNN configurations. For CNN_1_, Pearson’s correlation coefficient (r) and RMSE were equal to 0.86 and 0.27 mmHg/ml, respectively. The performance was found to be greatly improved for CNN_2_ (*r* = 0.97 and RMSE = 0.13 mmHg/ml). Moreover, all absolute errors from CNN_2_ were found to be less than 0.5 mmHg/ml. Importantly, the brachial BP wave appeared to be a promising source of information for estimating E_*es*_. Predictions were found to be in good agreement with the reference E_*es*_ values over an extensive range of LV contractility values and loading conditions. Therefore, the proposed methodology could be easily transferred to the bedside and potentially facilitate the clinical use of E_*es*_ for monitoring the contractile state of the heart in the real-life setting.

## 1 Introduction

Left ventricular (LV) contractility is a major determinant of the cardiac systolic function, ventricular-arterial interaction (Suga et al., 1973; Sagawa et al., 1977) as well as hemodynamical state (Cecconi et al., 2014). Currently, the gold standard method for evaluating LV systolic function is the invasive measurement of LV pressure-volume loops under varying load conditions, whereby the end-systolic pressure-volume relation (ESPVR) is derived (Suga et al., 1973; Suga and Sagawa, 1974; Sagawa et al., 1977). The ESPVR, described by its slope, i.e., the end-systolic elastance (E_*es*_), and its intercept, i.e., the dead volume (V_*d*_), has been proved to be less load sensitive than other indices of ventricular contractility (Paley et al., 1971). For an increased value of E_*es*_, the left ventricle is able to eject a higher blood volume against the same afterload, which is indicative of increased contractility (Suga and Sagawa, 1974). Evaluation of E_*es*_ is of utmost significance in clinical practice. The age-induced vascular stiffening (Chen et al., 1998) and hypertension (Borlaug et al., 2009) are strongly associated to the stiffening of the left ventricle, which is followed by an increase in E_*es*_. Furthermore, continuous and reliable monitoring of E_*es*_ is critical in patients with heart failure or septic cardiomyopathy (Cecconi et al., 2014). Yet, the bedside use of E_*es*_ is not established due to the invasive nature and high costs of the existing measuring techniques (Sagawa, 1981). Such limitations create an inescapable need for a new method that will permit the E_*es*_ derivation in a fast, simple, non-invasive manner using easily obtained measurements (such as applanation tonometry).

Arterial pulse waves contain a wealth of information for assessing the cardiovascular health in humans. Importantly, the morphology of the arterial pulse is affected by the mechanical and structural properties of the heart and the arterial network (Charlton et al., 2019). Clinical studies have investigated the arterial hemodynamics in normal and diseased human hearts under varying loading conditions and inotropic states, showing that the shape of the arterial BP waveform is highly sensitive to changes in LV E_*es*_ (Mikulic et al., 1977). Interestingly, Ostadal et al. have presented data verifying that continuous monitoring of dP/dt_max_ (where BP time-signal is measured *via* arterial line) enables the assessment of the LV function in patients with acute heart failure (Ostadal et al., 2019). In particular, the dP/dt_max_ can be calculated from a BP waveform, obtained either minimally invasively from a peripheral arterial line (De Hert et al., 2006; Morimont et al., 2012; Garcia et al., 2018) or non-invasively using, for instance, a tonometry-based device (Tartiere et al., 2007). Nonetheless, there is no current study to investigate the importance of exploiting the entire BP waveform (time sequence and its time derivative) for further facilitating the non-invasive monitoring of LV contractility.

Recent advancements in the field of artificial intelligence have introduced novel methods towards the predictive modelling for clinical use, creating a promising opportunity for further methodological advancements (Ramesh et al., 2004). Yet, only few studies have leveraged machine learning and deep learning techniques for cardiac monitoring (Huttunen et al., 2020; Bikia et al., 2020, 2021). Motivated by the evidence provided by the current state of knowledge, the present study aims to explore the opportunity in using the entire brachial BP wave for predicting LV E_*es*_ *via* convolution neural networks (CNNs). The requirement of a broad training dataset is addressed by the use of an in silico cohort, which was generated by a validated one-dimensional (1-D) cardiovascular simulator (Reymond et al., 2009). In silico models permit studying and understanding of various pathophysiological conditions, whereas they provide additional hemodynamic insights, which would be difficult to obtain *in vivo*. Concurrently, accurate measurement of E_*es*_ is challenging in a human cohort and thus a preliminary in silico verification of the proposed concept would benefit the future *in vivo* validation. Our aim was to propose an original conceptual methodology for continuous monitoring of the cardiac performance and to evaluate its feasibility in silico. The result of the in silico experiments can be considered as preliminary implications for the accuracy of the predictions under ideal conditions.

## 2 Materials and Methods

### 2.1 Brief Description of the 1-D Cardiovascular Model

**FIGURE 1:**
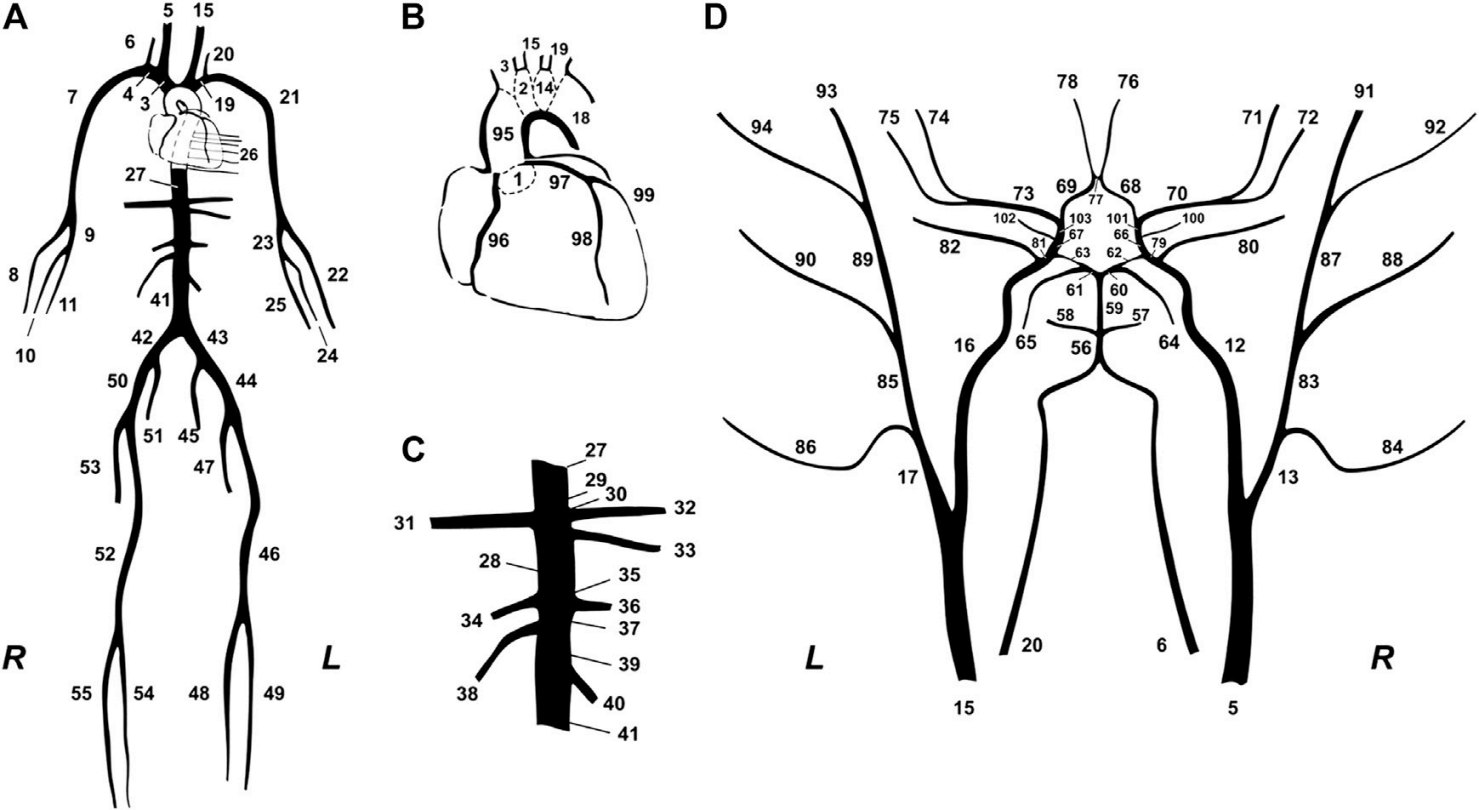
Schematic representation of the model of systemic circulation developed by (Reymond et al., 2009). **(A)** Main systemic arterial tree. **(B)** Detail of the aortic arch and the coronary network. **(C)** Detail of the principal abdominal aorta branches. **(D)** Blown-up schematic of the detailed cerebral arterial tree, which is connected *via* the carotids (segments 5 and 15) and the vertebrals (segments 6 and 20) to the main arterial tree shown in **(A)**.

We adopted a 1-D mathematical model of the cardiovasculature (Figure 1) which has been previously described in (Reymond et al., 2009). The arterial tree network includes all major vessels of the systemic circulation, as well as the cerebral circulation and the coronary circulation. The governing equations of the model are derived by integrating the longitudinal momentum and continuity of the Navier-Stokes equations over the arterial cross-section. The models solves the governing equations with proper boundary conditions and provides flow and pressure at every arterial location of the network. Every arterial segment is modelled as a long, tapered tube, and its compliance is defined as a non-linear function of pressure and location (Langewouters, 1984). Terminal vessels are coupled with three-element Windkessel models (Westerhof et al., 2009) and intimal shear is modelled following the Witzig-Womersley theory (Womersley, 1957). At the proximal end (at the root of the aorta), the arterial tree is coupled with a time-varying elastance model (VEM) of the left ventricle (Suga and Sagawa, 1974; Sagawa et al., 1977). Specifically, the VEM simulates the relationship between the LV pressure (P_*LV*_) and LV volume (V_*LV*_), namely:

$$E\left(t\right)=\frac{P_{LV}}{V_{LV}-V_{d}}$$ (1)

where V_*d*_ is the LV dead volume. Table 1 summarizes all the inputs and outputs of the 1-D cardiovascular model. A detailed description of the 1-D simulator can be found in the original publications (Reymond et al., 2009, Reymond et al., 2011).

**TABLE 1:** List of inputs and outputs of the 1-D cardiovascular model.

	**Corresponding variable**	**Value**
**Inputs**		
End-systolic elastance (mmHg/ml)	Ees	2.6
End-diastolic elastance (mmHg/ml)	Eed	0.08
Filling pressure (mmHg)	Pfill	14
Time of maximal elastance (ms)	tes	340
Heart rate (bpm)	HR	75
Dead volume (ml)	Vd	15
Venous resistance (mmHg.s/ml)	Rven	0.003
Arterial distensibility (10^–3^/mmHg)	C	(no_segments)x1 vector
Terminal compliances (ml/mmHg)	Ct	(no terminal segments)x1 vector
Peripheral resistances (mmHg.s/ml)	Rt	(no terminal segments) x1 vector
Arterial inlet diameter (cm)	din	(no_segments)x1 vector
Arterial outlet diameter (cm)	dout	(no_segments)x1 vector
Arterial length (cm)	len	(no_segments)x1 vector
Blood density (kg/m^3^)	ρ	1,050
Blood viscosity [Pa.s)	μ	0.004
**Outputs**		
Pressure waves (mmHg)	pressures	(no segments)x(no time points) vector
Flow waves (ml/s)	flows	(no segments)x(no time points) vector

### 2.2 Description of the in Silico Dataset

For generating various hemodynamic cases, the 1-D cardiovascular simulator ran using different combinations of arbitrary input model parameters. The distributions of the input model parameters were based on literature data, by identifying the normal values and ranges of the parameters. Given that the literature data are only provided in terms of mean and standard deviation or/and minimum and maximum values, the exact distribution of each parameter was unknown. In addition, varying the parameters while accounting for dependencies between parameters was not feasible due to the lack of sufficient data to inform inter-dependencies. Therefore, the sampling was selected to be random Gaussian.

The selected distributions of the input model parameters are summarized in Table 2. The parameters of arterial distensibility and terminal compliance were altered simultaneously, while nonuniform aortic stiffening was considered for the elderly and hypertensive virtual subjects, following the methodology described in our previous work (Bikia et al., 2019; Pagoulatou et al., 2019). Peripheral resistances were modified uniformly in order to achieve the specific value of total peripheral resistance in the selected range.

**TABLE 2:** Selected distributions of the model’s input parameters based on the literature.

**Parameter**	**mean ± SD**	**References**
End-systolic elastance (mmHg/ml)	2.3 ± 1	Chen et al. (1998)
End-diastolic elastance (mmHg/ml)	0.2 ± 0.11	Chen et al. (1998)
Filling pressure (mmHg)	15 ± 5.4	Senzaki et al. (1996)
Time of maximal elastance (ms)	327 ± 39	Starling et al. (1987)
Heart rate (bpm)	63.7 ± 9.5	Segers et al. (2008)
Aortic distensibility (10^–3^/mmHg)	5.86 ± 3.23	Dogui et al. (2011)
Total peripheral resistance (mmHg.s/ml)	1.28 ± 0.31	Segers et al. (2008)
Aortic diameter (cm)	33.2 ± 4.1	Wolak et al. (2008)
Height (cm)	169.2 ± 8.9	Segers et al. (2008)

Furthermore, the geometry of the arterial network (namely length, inlet diameter, and outlet diameter of the arterial segments) was modified to simulate different body types by adapting the length and the diameter of all arterial vessels. The reference state of the arterial tree model corresponds to an individual with a height equal to 180 cm. Different heights were simulated *via* multiplication of the reference arterial lengths with a scaling factor (uniform adaptation). As per the arterial diameters, previous studies have associated the variation of the aortic diameter with respect to age, gender, weight, and height (Wolak et al., 2008). However, there exist no sufficient available data to demonstrate the diameter variation of multiple arterial segments with respect to an individual’s demographic profile. As a result, we modified all arterial segments following a uniform distribution based on the variation of the aortic diameter.

In order to eliminate the likelihood of creating unrealistic hemodynamical profiles, we examined the physiological validity of every case and discarded any implausible generated virtual subject. The physiological validity of each subject was evaluated by comparing the simulated brachial and aortic BP values [i.e., SBP, DBP, MAP, and pulse pressure (PP)] to the reference values reported in the literature (McEniery et al., 2005). A subject was discarded from the data if any of the BP values did not lie within the range of mean ±2.807SD (assuming 99.5% confidence intervals). For deriving the dataset, we ran the model 10,000 times to generate 10,000 cases. Out of the 10,000 cases, 3,748 samples were accepted after applying the above filtering criteria.

### 2.3 Data Pre-processing

The brachial BP waveform was derived from the left simulated brachial artery. The train/validation/test split was set to be 60% (2,248 cases)/20% (750 cases)/20% (750 cases). By computing the MSE with decreasing training size, we noticed that similar results can be achieved with fewer samples (e.g., 1,603) and, therefore, we may deduce that a training size of 2,248 is sufficient.

The BP waveforms were up-sampled so that each wave consists of 200 samples. This selection allowed us to ensure a sampling frequency higher than the 100-Hz threshold suggested for the pulse wave velocity techniques (Gaddum et al., 2013) (which require substantially high signal resolution). This value was considered as a fair trade-off between computational time and high signal fidelity.

Subsequently, the data were normalized using the MinMaxScaler () function from Sklearn library. The Min-max normalization method is a standard normalization approach which guarantees that all features are on the same scale, e.g., between zero and one. Other methods, such as the z-score or feature clipping, are preferable when there are several outliers in the data. Given that the filtering of the in silico population essentially disregards the outliers, the Min-max method may be sufficient for our learning algorithm.

### 2.4 Convolution Neural Networks

We evaluated two model configurations with respect to the inputs:

1. One-channel CNN (CNN_1_): Using as a sole input the entire BP waveform.
2. Two-channels CNN (CNN_2_): Using as inputs the entire BP waveform and its time derivative.

The time derivative of the BP wave was calculated as the slope of the wave using the central differences approach:

$$f^{\prime }\left[n\right]=\frac{f\left[n+1\right]-f\left[n-1\right]}{2\tau }$$ (2)

where *f* [*n*] is the BP function at the *n^th^* time point and *τ* is the time interval between the two pressure values. The *τ* is computed as the entire heart cycle duration divided by the number of recorded pressure values (200 samples).

The CNN models were created using PyTorch library (Paszke et al., 2019). In particular, the networks were composed of four 1-D Convolutional layers, each of them followed by an activation ReLU layer. Following the four convolutional layers intercalated with the activation ReLU layers, three additional functions were used to yield the final output results. Firstly, we employed a MaxPooling layer which uses the MaxPool1d function from PyTorch framework. The MaxPooling function permits to progressively reduce the spatial size of the data for keeping only the maximum of each window while striding (kernel_size = 3, stride = 2). The MaxPooling layer was followed by a Flatten function which flattened the output of the convolutional layers to create a single long feature vector. A Linear layer was finally applied on the output of the Flatten function, providing the final prediction of the E_*es*_ value. The functions are further described in the torch. nn module (Available at: https://pytorch.org/docs/stable/generated/torch.nn).

In order to generate our different CNN models, we made use of PyTorch Conv1D () function with different values for in_channels and out_channels parameters. The input data size was 200 for CNN_1_ and 200 × 2 for CNN_2_. In addition, the kernel size of each filter was set to 5, which is a popular choice in the state of the art. Importantly, we opted for an odd-sized filter, as all the previous layer pixels would be symmetrically around the output pixel. Selecting even-sized kernel filters would require us to account for distortions across the layers. Therefore, odd-sized kernel filters were preferred for implementation simplicity. The value of stride and padding was kept constant throughout the models and equal to 2.

**FIGURE 2:**
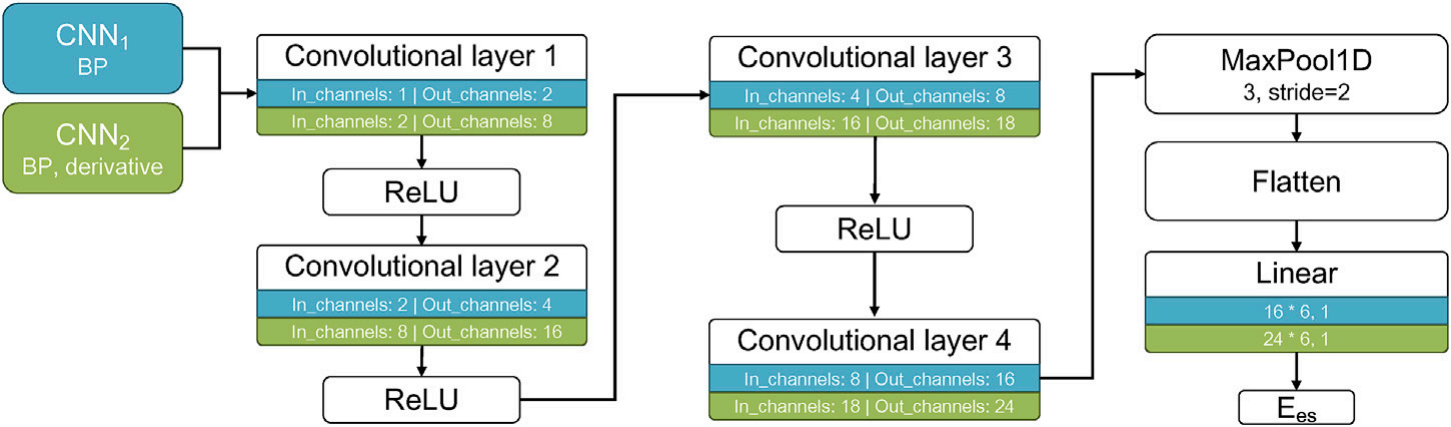
Architecture of the CNN models. The two CNN models are shown in different colors with their respective inputs listed, as well as the number of in_channels and out_channels for each convolutional layer and the output E_*es*_.

Each of the CNN model with each own input layer was characterized by the respective number of channels. Figure 2 illustrates the number of inputs/outputs between each convolutional layer, and the architecture of the two models. The number of filters per channel on each convolutional layer is presented in Table 3. The number of filters was optimized by an “error and trial” approach, and the optimal values were selected for the specific type of data.

**TABLE 3:** Number of filters per each convolutional layer for the two CNN models.

	**Number of filters per channel**	**Total (no filters x no input channels)**
**CNN**_**1**_		
Layer 1	2	2
Layer 2	4	8
Layer 3	8	32
Layer 4	16	128
**CNN**_**2**_		
Layer 1	8	16
Layer 2	16	128
Layer 3	18	288
Layer 4	24	432

The CNN parameters, namely the weights and biases, were optimized upon training on 60% of the dataset. The resulting model was then applied to the validation set (20% of the whole dataset) in order to assess the loss and the accuracy. On this validation set, we performed tuning for two hyperparameters, namely the batch_size and the number of epochs. This allowed us to ensure that no overfitting occurred. The value of learning_rate was set equal to 0.001 and tuning was performed using the Adam Optimizer (Kingma and Ba, 2017) for batch_size values (32, 64, 128) and epochs values within the range of (1, 400). Adam is a versatile optimization method. Given the satisfactory performance of our trained models, we did not consider evaluating additional algorithms.

The trained CNN models using the tuned hyperparameters along with the weights and biases values were applied to the test set (remaining 20% of the data) in order to evaluate the predictive performance of the models. The tuning process was conducted with regard to the mean square error (MSE) loss function. The MSE loss function is considered as a fair selection under the inference framework of maximum likelihood when the distribution of the target variable is Gaussian-like (as in the present study). In addition, it is preferable in comparison to other methods which might be more computationally expensive (e.g. the mean absolute error method which uses modulus operator function) or might impose increased training requirements (e.g. the uber loss which involves the optimization of the hyperparameter *δ* in order to maximize model accuracy).

### 2.5 Sensitivity to Errors

**FIGURE 3:**
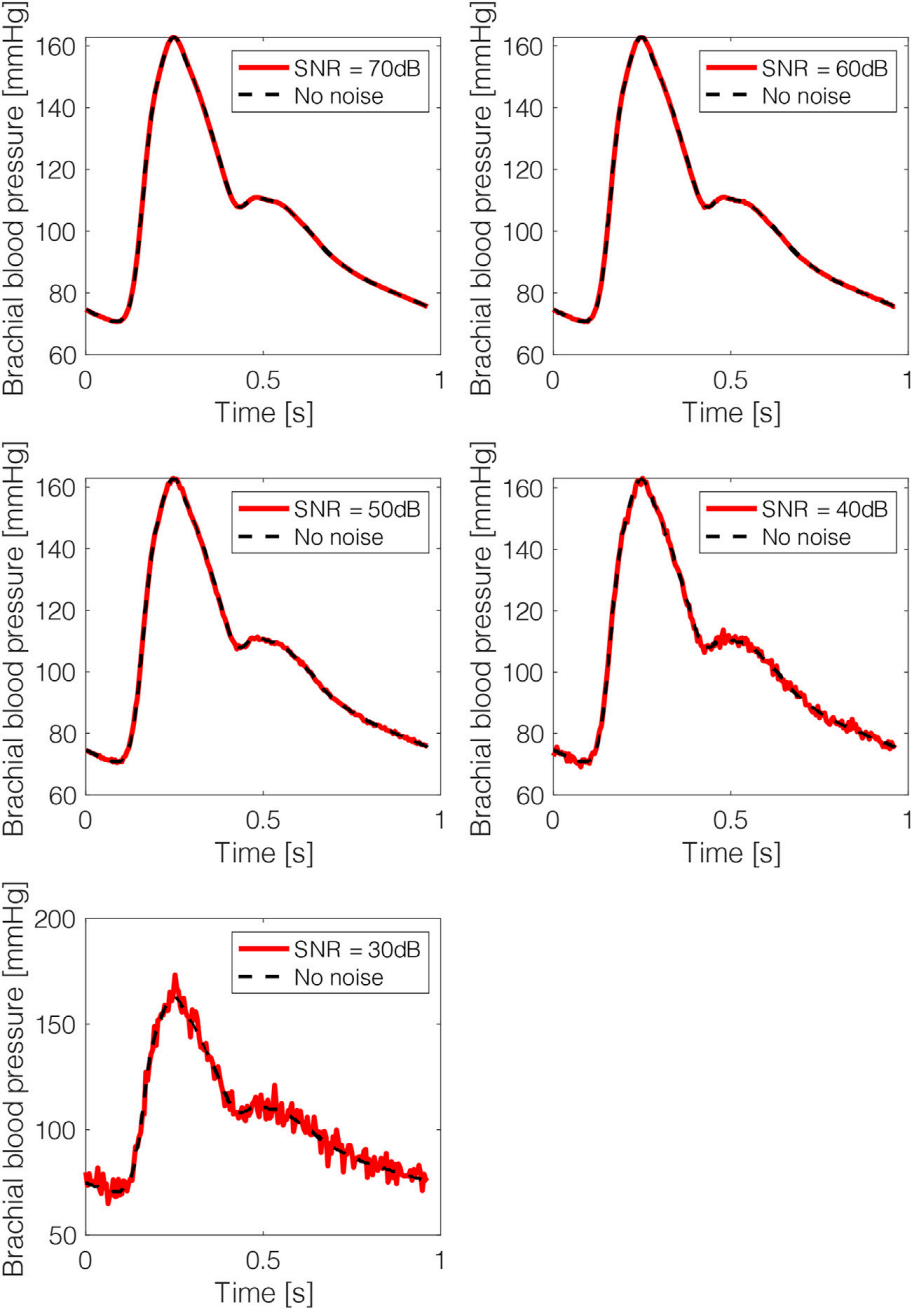
Brachial blood pressure waves after adding artificial noise. The noisy data are presented in red solid lines and the original noise-free data in black dashed lines.

In order to investigate the impact of potential errors or adverse effects in the measurements of the BP signal, the test data were corrupted with artificial noise. White gaussian noise (WGN) was added to the BP for each subject using the awgn () MATLAB function (The Math Works, Inc. MATLAB. Version 2020b). The performance of the two CNN models was tested for five values of signal-to-noise ratio (SNR), i.e. 70, 60, 50, 40, 30 dB. The metrics of agreement and accuracy were reported for each level of noise. Examples of the noise effect on the BP wave are depicted in Figure 3.

### 2.6 Statistical Analysis

The performance of the models in terms of agreement, bias and accuracy, was evaluated with the use of the Pearson’s correlation coefficient (*r*), the normalized root mean square error (nRMSE), and the Bland-Altman analysis (Bland and Altman, 2010). The computed nRMSE was based on the difference between the minimum and maximum values of the dependent variable. A *p*-value below 0.05 was considered as statistically significant. The statistical analysis was performed in Python (Python Software Foundation, Python Language Reference, version 3.6.8, Available at http://www.python.org).

## 3 Results

Table 4 presents the cardiac and vascular characteristics of the study population (3,748 cases). The CNN-derived E_*es*_ were compared to the reference E_*es*_ values, which were provided by the 1-D cardiovascular model.

**TABLE 4:** Cardiovascular characteristics of the virtual study cohort (*n* = 3,748).

**Parameter**	**Mean ± SD (*n* = 3,748)**
End-systolic elastance (mmHg/ml)	2.4 ± 0.52
End-diastolic elastance (mmHg/ml)	0.16 ± 0.04
Filling pressure (mmHg)	16.54 ± 3.19
Time of maximal elastance (ms)	328 ± 23
Heart rate (bpm)	75.96 ± 8.25
Ejection fraction (%)	47.38 ± 6.06
Stroke volume (ml)	56.68 ± 12.75
Aortic systolic blood pressure (mmHg)	110.62 ± 23.13
Aortic diastolic blood pressure (mmHg)	80.93 ± 14.79
Aortic pulse pressure (mmHg)	29.70 ± 13.04
Mean arterial pressure (mmHg)	95.71 ± 18.40
Brachial systolic blood pressure (mmHg)	121.64 ± 24.07
Brachial diastolic blood pressure (mmHg)	78.71 ± 14.44
Brachial pulse pressure (mmHg)	42.93 ± 15.05
Pulse pressure amplification	1.49 ± 0.11
Total peripheral resistance (mmHg.s/ml)	1.36 ± 0.17
Total arterial compliance (ml/mmHg)	1.27 ± 0.41

### 3.1 Comparison Between the CNN Predicted E_*es*_ and the Reference E_*es*_ Values

**FIGURE 4:**
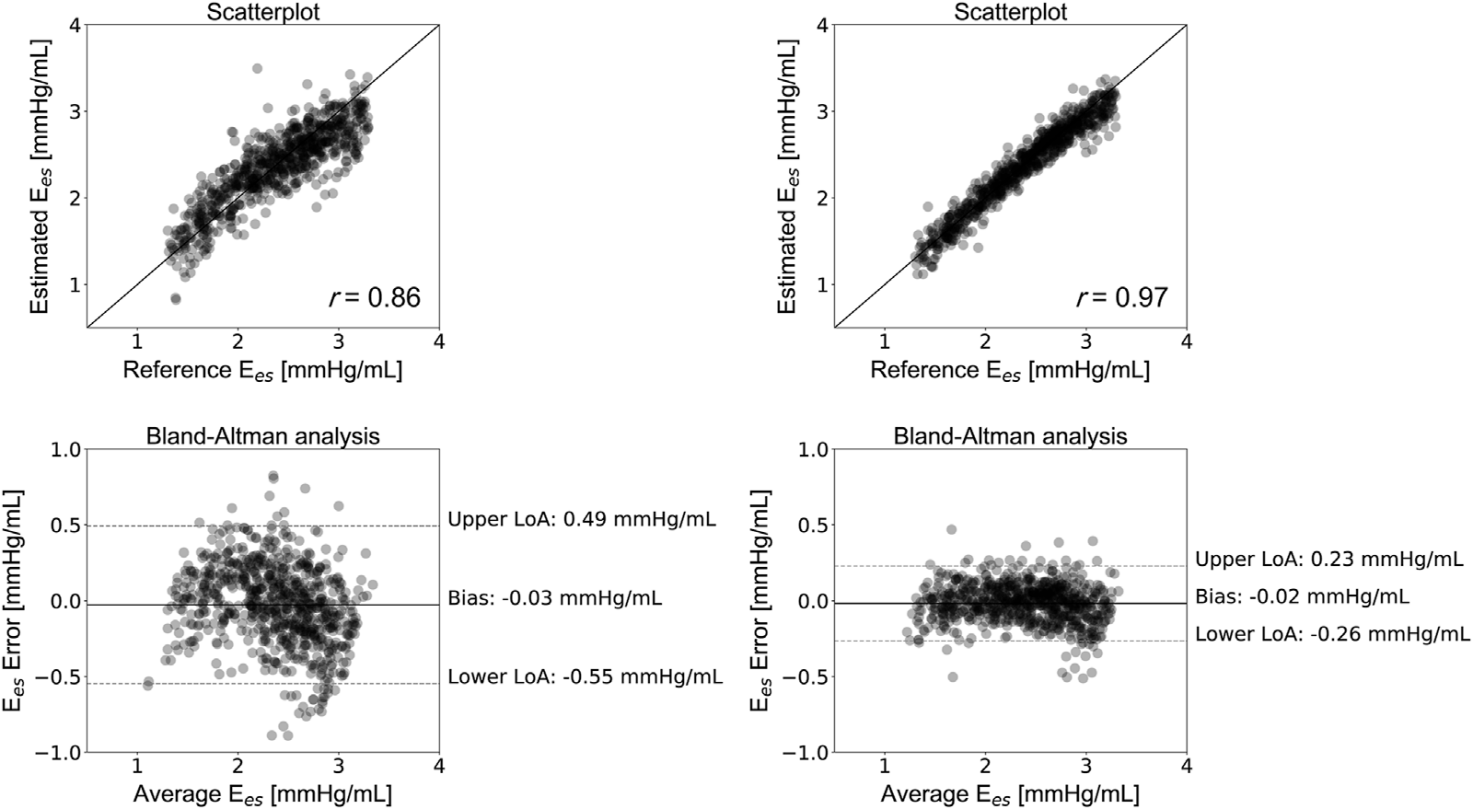
Comparison between predicted and reference elastance data. Scatterplots and Bland–Altman plots between the predicted E_*es*_ and the reference E_*es*_ for CNN_1_ **(left panel)** and CNN_2_ **(right panel).** The solid line of the scatterplots represents equality. In Bland–Altman plots, limits of agreement (LoA), within which 95% of errors are expected to lie, are defined by the two horizontal dashed lines.

Table 5 summarizes the regression metrics of the statistical comparisons between the non-invasive E_*es*_ estimates and the reference E_*es*_. The Bland-Altman analysis indicated a low bias for the estimated E_*es*_. The limits of agreement (LoA) between the estimated and reference E_*es*_ (within which 95% of errors are expected to lie) were found to be (−0.55, 0.49) mmHg/ml and (−0.26, 0.23) mmHg/ml, for CNN_1_ and CNN_2_, respectively. Figure 4 illustrates the scatterplots and the Bland-Altman plots of the estimated E_*es*_ against the actual E_*es*_. The absolute difference between the estimated E_*es*_ and the real E_*es*_ values did not exceed 0.5 mmHg/ml in 95% of the total test cases for CNN_1_, while all errors were found to be smaller than 0.5 mmHg/ml for CNN_2_. Furthermore, for the CNN_2_ configuration, the absolute error was less than 0.05 mmHg/ml in 61% of the test set.

**TABLE 5:** Regression statistics between model-predicted and reference elastance values.

**Model**	**SNR (dB)**	**Slope**	**Intercept (mmHg/ml)**	**r**	***p*** **-value**	**nRMSE (%)**	**Bias (LoA) (mmHg/ml)**	**Predicted E_*es*_ (mmHg/ml)**
CNN_1_	No noise	0.75	0.56	0.86		13.4	−0.03 (−0.55, 0.49)	2.36 ± 0.45
	70	0.75	0.56	0.86		13.4	−0.03 (−0.55, 0.49)	2.36 ± 0.45
	60	0.75	0.56	0.86		13.4	−0.03 (−0.54, 0.49)	2.36 ± 0.45
	50	0.76	0.56	0.85		13.5	−0.02 (−0.55, 0.5)	2.36 ± 0.45
	40	0.75	0.57	0.83		14.7	−0.03 (−0.6, 0.55)	2.36 ± 0.46
	30	0.72	0.66	0.61		25.2	0.00 (−0.98, 0.98)	2.39 ± 0.61
CNN_2_	No noise	0.94	0.12	0.97	<0.0001	6.4	−0.02 (−0.26, 0.23)	2.37 ± 0.5
	70	0.94	0.12	0.97		6.5	−0.02 (−0.27, 0.23)	2.37 ± 0.5
	60	0.94	0.11	0.96		7.3	−0.02 (−0.31, 0.26)	2.36 ± 0.5
	50	0.93	0.12	0.88		13.5	−0.04 (−0.56, 0.47)	2.34 ± 0.54
	40	0.88	0.47	0.59		32.9	0.2 (−1.04, 1.42)	2.57 ± 0.77
	30	0.87	2.76	0.29		144	2.45 (−0.45, 5.6)	4.84 ± 1.55

SNR: signal-to-noise ratio; r: Pearson’s correlation coefficient; nRMSE: normalized root mean square error; LoA: limits of agreement.

Two-sided P-value for a hypothesis test whose null hypothesis is that the slope is zero, using Wald Test with t-distribution of the test statist

The computational time required for training the models was 110 and 115 s for CNN_1_ and CNN_2_, respectively. The time required to yield the predictions for the test set was reported to be less than 1 s.

### 3.2 Sensitivity to Errors

The impact of potential errors or adverse effects in the measurements of the BP signal was quantified for the two CNN configurations under various noise levels (Table 3). The CNN_1_ model appeared to be robust for an SNR value equal or larger than 40 dB (nRMSE <15%). On the other hand, the performance of CNN_2_ remained unaffected for SNR ≥60 dB (nRMSE was doubled for higher values of SNR). However, when the SNR reduced to 40 dB or less, the correlation and agreement were significantly deteriorated (r < 0.6 and nRMSE >30%).

## 4 Discussion

In the present study, we suggested that the prediction of the cardiac contractility index of E_*es*_ is feasible using a single brachial BP waveform. The proposed concept was appraised using an in silico dataset which was generated using a 1-D mathematical model of the cardiovascular system (Reymond et al., 2009). The results showed that the brachial BP wave may be valuable for the characterization of E_*es*_. In particular, the CNN configuration combining the brachial BP wave and its time derivative provided higher precision than the precision achieved by the CNN that used only the BP signal (correlation was increased from 0.86 to 0.97).

Arterial pulse wave contains a wealth of physiological information as its morphology is influenced by the heart and the systemic circulation (Charlton et al., 2019). Quantities such as stroke volume as well as the arterial stiffness and wave reflections have a prominent impact on the arterial pulse. Furthermore, pathological changes affect the arterial pulse in different ways, including the amplitude, shape, and frequency (Westerhof et al., 2018). As a result, arterial pulse waves provide abundant and reliable information about the cardiovascular function. Importantly, physiological parameters derived from the arterial pulse can be useful for diagnosis and clinical decision making. Arterial waves can be easily measured using non-invasive clinical devices, such as oscillometric or tonometric BP monitors. In addition, arterial waves from photoplethysmography (PPG) or other signals including the electrocardiogram (ECG), are also routinely monitored by wearable devices (e.g. smartwatches and fitness wristbands). Hence, the high accessibility of the arterial pulse waves in both clinical settings and daily life encourages further exploitation of their insights with respect to the cardiovascular function.

With the increasing availability of clinical data, signals, and images sourced from various avenues of medicine and healthcare, the application of artificial intelligence for analysis and interpretation of medical data grows rapidly. The diagnosis of the cardiovascular disease could benefit essentially from early prediction, prevention, and proactive management. Thus artificial intelligence-based methodologies could essentially contribute towards this direction. Deep learning offers a promising potential in exploring new methods for cardiac monitoring by deciphering key information in arterial waveforms. Deep learning is a class of machine learning algorithms that uses multiple layers to progressively extract higher-level features from the raw input. In this study, we leveraged this exact capacity of CNN models in order to evaluate LV E_*es*_ from a single BP waveform. Such potential can open new directives in digital health and potentially suggest new markers for cardiac monitoring purposes.

Ensuring high fidelity in the signal acquisition constitutes a critical aspect for the accurate estimation of E_*es*_. Especially, caution should be paid in successfully capturing the waveform, as the measurement may be prone to errors or adverse effects which can distort the relevant information for the deep CNN prediction. In order to evaluate the effect of errors in the morphology of the input brachial BP wave, we artificially introduced simulated noise. The noise was applied only on the test set which was subsequently fed to the trained CNN models. The sensitivity analysis showed that subtle distortion in the wave shape did not significantly affect the accuracy of the CNN models. However, the performance was severely worsened when the SNR approached 30 dB. The CNN_1_ was found to be more robust to measurement noise when compared to the CNN_2_ whose estimation relies on both the pressure wave and its time derivative. This might be explained by the sensitivity of the CNN_2_ to two input waves. Specifically, the error may propagate through the derivative computation by directly altering the two derivative factors (i.e., f [n-1] and f [n+1]) and, subsequently, influence to a greater extent the deep CNN prediction.

Previous methods on the estimation of E_*es*_ rely mainly on non-invasive single-beat measurements (Shishido et al., 2000; Chen et al., 2001; Bikia et al., 2020; Pagoulatou et al., 2021). These methods require the inclusion of cuff BP, stroke volume, ejection fraction or other measurements. Especially, stroke volume and ejection fraction constitute common measures of the LV systolic function and can be obtained *via* several cardiac imaging modalities, such as the magnetic resonance imaging, and the Simpson’s method. However, these imaging techniques are tedious and require a highly trained technician. In addition, ejection fraction expresses the stroke volume as a fraction of end-diastolic volume (EDV), and, therefore, correct interpretation of ejection fraction can be achieved only with the additional knowledge of EDV. Simplification of the E_*es*_ approximation by using a sole BP wave recording may facilitate cardiac monitoring while reducing costs and complexity for the clinicians and the patients.

It is to be highlighted that this study aimed to address an unmet clinical need by proposing a novel methodology, dissimilar to the existing state of the art. As a result, there was not sufficient relevant literature to guide the CNN design and architecture for the research question under investigation. In particular, there did not exist previously published studies that aimed to address a similar problem and which could inform us about the selection of the model functions and parameters. Therefore, we developed and suggested an original architecture that fits best in the specific type of data.

Several limitations of the present study need to be acknowledged. The current study was entirely based on simulated data and thus the results should be considered as a preliminary assessment of the theoretical concept of the proposed approach. While synthetic data can mimic numerous properties of the real clinical data, they do not copy the original content in an identical way. Future work should include the use of real clinical data that will finally verify the application of the proposed method in the clinical setting. It is likely that the models trained using the in silico data are not capable for adequate predictions using real human data. Nevertheless, in silico trained networks could be used in transfer learning as pre-trained networks which are subsequently fine tuned with clinical measurements. At this stage of our research, we found it reasonable to start with an in silico validation of our research hypothesis, instead of directly collecting measurements of E_*es*_ in humans. The cost and the complexity of the E_*es*_ measurements would make it difficult to incorporate them in the current study. In addition, the variance of the simulated ejection fraction data was reported to be low, while the average ejection fraction was equal to 47%. Such a data distribution represents more accurately a population with heart problems. Our future *in vivo* studies will include a wider range of ejection fraction values, which will account for both diseased and healthy populations. Finally, the evaluation of the proposed framework was done using a single beat of each virtual subject. Next steps will also include the in silico and the *in vivo* validation of a CNN method that uses multiple heart beats from every participant. Hence, a closed-loop cardiovascular mathematical model may be adopted for achieving this goal.

## 5 Conclusion

We showed that the use of the brachial BP waveform in conjunction with a deep CNN provided accurate estimates of E_*es*_. In particular, our findings indicated that the brachial BP wave may be a promising source of information for assessing E_*es*_ and its clinical utility should be emphasized. Our prediction algorithm achieved a satisfactory performance for an extensive range of LV contractility values and loading conditions. Consequently, the proposed methodological concept could be readily transferred to the bedside and potentially enhance the clinical use of E_*es*_ for monitoring the contractile state of the heart in the real-life medical environment.

## Data Availability

The raw data supporting the conclusions of this article will be made available by the authors, without undue reservation.

## References

[B1] Bikia V. Papaioannou T. G. Pagoulatou S. Rovas G. Oikonomou E. Siasos G. (2020). Noninvasive Estimation of Aortic Hemodynamics and Cardiac Contractility Using Machine Learning. Sci. Rep. 10, 15015–15017. 10.1038/s41598-020-72147-8 32929108 PMC7490416

[B2] Bikia V. Adamopoulos D. Pagoulatou S. Rovas G. Stergiopulos N. (2021). Ai-based Estimation of End-Systolic Elastance from Arm-Pressure and Systolic Time Intervals. Front. Artif. Intell. 4, 16. 10.3389/frai.2021.579541 PMC8079739 33937742

[B3] Bikia V. Pagoulatou S. Trachet B. Soulis D. Protogerou A. Papaioannou T. (2019). Noninvasive Cardiac Output and central Systolic Pressure from Cuff-Pressure and Pulse Wave Velocity: A Model-Based Study. IEEE J. Biomed. Health Inform. 24 (7), 1968–1981. 10.1109/jbhi.2019.2956604 31796418

[B4] Bland J. M. Altman D. G. (2010). Statistical Methods for Assessing Agreement between Two Methods of Clinical Measurement. Int. J. Nurs. Stud. 47, 931–936. 10.1016/j.ijnurstu.2009.10.001 2868172

[B5] Borlaug B. A. Lam C. S. P. Roger V. L. Rodeheffer R. J. Redfield M. M. (2009). Contractility and Ventricular Systolic Stiffening in Hypertensive Heart Disease. J. Am. Coll. Cardiol. 54, 410–418. 10.1016/j.jacc.2009.05.013 19628115 PMC2753478

[B6] Cecconi M. De Backer D. Antonelli M. Beale R. Bakker J. Hofer C. (2014). Consensus on Circulatory Shock and Hemodynamic Monitoring. Task Force of the European Society of Intensive Care Medicine. Intensive Care Med. 40, 1795–1815. 10.1007/s00134-014-3525-z 25392034 PMC4239778

[B7] Charlton P. H. Mariscal Harana J. Vennin S. Li Y. Chowienczyk P. Alastruey J. (2019). Modeling Arterial Pulse Waves in Healthy Aging: a Database for In Silico Evaluation of Hemodynamics and Pulse Wave Indexes. Am. J. Physiology-Heart Circulatory Physiol. 317, H1062–H1085. 10.1152/ajpheart.00218.2019 PMC6879924 31442381

[B8] Chen C.-H. Fetics B. Nevo E. Rochitte C. E. Chiou K.-R. Ding P.-A. (2001). Noninvasive Single-Beat Determination of Left Ventricular End-Systolic Elastance in Humans. J. Am. Coll. Cardiol. 38, 2028–2034. 10.1016/s0735-1097(01)01651-5 11738311

[B9] Chen C.-H. Nakayama M. Nevo E. Fetics B. J. Maughan W. L. Kass D. A. (1998). Coupled Systolic-Ventricular and Vascular Stiffening with Age. J. Am. Coll. Cardiol. 32, 1221–1227. 10.1016/s0735-1097(98)00374-x 9809929

[B10] De Hert S. G. Robert D. Cromheecke S. Michard F. Nijs J. Rodrigus I. E. (2006). Evaluation of Left Ventricular Function in Anesthetized Patients Using Femoral Artery Dp/dtmax. J. Cardiothorac. Vasc. Anesth. 20, 325–330. 10.1053/j.jvca.2005.11.006 16750731

[B11] Dogui A. Kachenoura N. Frouin F. Lefort M. De Cesare A. Mousseaux E. (2011). Consistency of Aortic Distensibility and Pulse Wave Velocity Estimates with Respect to the Bramwell-hill Theoretical Model: a Cardiovascular Magnetic Resonance Study. J. Cardiovasc. Magn. Reson. 13, 11. 10.1186/1532-429x-13-11 21272312 PMC3038969

[B12] Gaddum N. R. Alastruey J. Beerbaum P. Chowienczyk P. Schaeffter T. (2013). A Technical Assessment of Pulse Wave Velocity Algorithms Applied to Non-invasive Arterial Waveforms. Ann. Biomed. Eng. 41, 2617–2629. 10.1007/s10439-013-0854-y 23817766

[B18] Garcia M. I. M. Jian Z. Settels J. J. Hunley C. Cecconi M. Hatib F. (2018). Performance Comparison of Ventricular and Arterial dP/dt _max_ for Assessing Left Ventricular Systolic Function during Different Experimental Loading and Contractile Conditions. Crit. Care 22 (1), 1–12. 10.1186/s13054-018-2260-1 30486866 PMC6262953

[B13] Huttunen J. M. J. Kärkkäinen L. Honkala M. Lindholm H. (2020). Deep Learning for Prediction of Cardiac Indices from Photoplethysmographic Waveform: A Virtual Database Approach. Int. J. Numer. Method Biomed. Eng. 36, e3303. 10.1002/cnm.3303 31886948

[B14] Kingma D. P. Ba J. (2017). Adam: A Method for Stochastic Optimization. San Diego, US.

[B15] Langewouters G. J. Wesseling K. H. Goedhard W. J. A. (1984). The Static Elastic Properties of 45 Human Thoracic and 20 Abdominal Aortas *In Vitro* and the Parameters of a New Model. J. Biomech. 17, 425–435. 10.1016/0021-9290(84)90034-4 6480618

[B16] McEniery C. M. Yasmin n. Hall I. R. Qasem A. Wilkinson I. B. Cockcroft J. R. (2005). Normal Vascular Aging: Differential Effects on Wave Reflection and Aortic Pulse Wave Velocity. J. Am. Coll. Cardiol. 46, 1753–1760. 10.1016/j.jacc.2005.07.037 16256881

[B17] Mikulic E. Cohn J. N. Franciosa J. A. (1977). Comparative Hemodynamic Effects of Inotropic and Vasodilator Drugs in Severe Heart Failure. Circulation 56, 528–533. 10.1161/01.cir.56.4.528 902377

[B19] Morimont P. Lambermont B. Desaive T. Janssen N. Chase G. D'Orio V. (2012). Arterial dP/dtmax Accurately Reflects Left Ventricular Contractility during Shock when Adequate Vascular Filling Is Achieved. BMC Cardiovasc. Disord. 12, 13. 10.1186/1471-2261-12-13 22380679 PMC3313844

[B20] Ostadal P. Vondrakova D. Krüger A. Janotka M. Naar J. (2019). Continual Measurement of Arterial dP/dtmax Enables Minimally Invasive Monitoring of Left Ventricular Contractility in Patients with Acute Heart Failure. Crit. Care 23, 364–368. 10.1186/s13054-019-2654-8 31752966 PMC6869259

[B21] Pagoulatou S. Rommel K.-P. Kresoja K.-P. von Roeder M. Lurz P. Thiele H. (2021). *In Vivo* application and Validation of a Novel Noninvasive Method to Estimate the End-Systolic Elastance. Am. J. Physiology-Heart Circulatory Physiol. 320, H1543–H1553. 10.1152/ajpheart.00703.2020 33606586

[B22] Pagoulatou S. Z. Bikia V. Trachet B. Papaioannou T. G. Protogerou A. D. Stergiopulos N. (2019). On the Importance of the Nonuniform Aortic Stiffening in the Hemodynamics of Physiological Aging. Am. J. Physiology-Heart Circulatory Physiol. 317, H1125–H1133. 10.1152/ajpheart.00193.2019 31538801

[B23] Paley H. W. McDonald I. G. Blumenthal J. Mailhot J. Modin G. W. (1971). The Effects of Posture and Isoproterenol on the Velocity of Left Ventricular Contraction in Man. J. Clin. Invest. 50, 2283–2294. 10.1172/jci106726 4938131 PMC292170

[B24] Paszke A. Gross S. Massa F. Lerer A. Bradbury J. Chanan G. (2019). “Pytorch: An Imperative Style, High-Performance Deep Learning Library,” in Advances in Neural Information Processing Systems (Red Hook, NY: Curran Associates, Inc.), Vol. 32, 8024–8035.

[B40] Ramesh A. N. Kambhampati C. Monson J. R. Drew P. J. (2004). Artificial Intelligence in Medicine. Ann. R. Coll. Surg. Engl. 86 (5), 334–338. 10.1308/147870804290 15333167 PMC1964229

[B25] Reymond P. Bohraus Y. Perren F. Lazeyras F. Stergiopulos N. (2011). Validation of a Patient-specific One-Dimensional Model of the Systemic Arterial Tree. Am. J. Physiology-Heart Circulatory Physiol. 301, H1173–H1182. 10.1152/ajpheart.00821.2010 21622820

[B26] Reymond P. Merenda F. Perren F. Rüfenacht D. Stergiopulos N. (2009). Validation of a One-Dimensional Model of the Systemic Arterial Tree. Am. J. Physiology-Heart Circulatory Physiol. 297, H208–H222. 10.1152/ajpheart.00037.2009 19429832

[B27] Sagawa K. Suga H. Shoukas A. A. Bakalar K. M. (1977). End-systolic Pressure/volume Ratio: a New index of Ventricular Contractility. Am. J. Cardiol. 40, 748–753. 10.1016/0002-9149(77)90192-8 920611

[B28] Sagawa K. (1981). The End-Systolic Pressure-Volume Relation of the Ventricle: Definition, Modifications and Clinical Use. Circulation 63, 1223–1227. 10.1161/01.cir.63.6.1223 7014027

[B29] Segers P. Rietzschel E. R. De Buyzere M. L. Stergiopulos N. Westerhof N. Van Bortel L. M. (2008). Three- and Four-Element Windkessel Models: Assessment of Their Fitting Performance in a Large Cohort of Healthy Middle-Aged Individuals. Proc. Inst. Mech. Eng. H 222, 417–428. 10.1243/09544119jeim287 18595354

[B30] Senzaki H. Chen C.-H. Kass D. A. (1996). Single-Beat Estimation of End-Systolic Pressure-Volume Relation in Humans. Circulation 94, 2497–2506. 10.1161/01.cir.94.10.2497 8921794

[B31] Shishido T. Hayashi K. Shigemi K. Sato T. Sugimachi M. Sunagawa K. (2000). Single-beat Estimation of End-Systolic Elastance Using Bilinearly Approximated Time-Varying Elastance Curve. Circulation 102, 1983–1989. 10.1161/01.cir.102.16.1983 11034949

[B32] Starling M. R. Walsh R. A. Dell'Italia L. J. Mancini G. B. Lasher J. C. Lancaster J. L. (1987). The Relationship of Various Measures of End-Systole to Left Ventricular Maximum Time-Varying Elastance in Man. Circulation 76, 32–43. 10.1161/01.cir.76.1.32 3594773

[B33] Suga H. Sagawa K. (1974). Instantaneous Pressure-Volume Relationships and Their Ratio in the Excised, Supported Canine Left Ventricle. Circ. Res. 35, 117–126. 10.1161/01.res.35.1.117 4841253

[B34] Suga H. Sagawa K. Shoukas A. A. (1973). Load independence of the Instantaneous Pressure-Volume Ratio of the Canine Left Ventricle and Effects of Epinephrine and Heart Rate on the Ratio. Circ. Res. 32, 314–322. 10.1161/01.res.32.3.314 4691336

[B35] Tartiere J.-M. Logeart D. Beauvais F. Chavelas C. Kesri L. Tabet J.-Y. (2007). Non-invasive Radial Pulse Wave Assessment for the Evaluation of Left Ventricular Systolic Performance in Heart Failure. Eur. J. Heart Fail. 9, 477–483. 10.1016/j.ejheart.2006.11.005 17254846

[B36] Westerhof N. Lankhaar J.-W. Westerhof B. E. (2009). The Arterial Windkessel. Med. Biol. Eng. Comput. 47, 131–141. 10.1007/s11517-008-0359-2 18543011

[B37] Westerhof N. Stergiopulos N. Noble M. I. Westerhof B. E. (2018). Snapshots of Hemodynamics: An Aid for Clinical Research and Graduate Education. Springer.

[B38] Wolak A. Gransar H. Thomson L. E. J. Friedman J. D. Hachamovitch R. Gutstein A. (2008). Aortic Size Assessment by Noncontrast Cardiac Computed Tomography: normal Limits by Age, Gender, and Body Surface Area. JACC: Cardiovasc. Imaging 1, 200–209. 10.1016/j.jcmg.2007.11.005 19356429

[B39] Womersley J. (1957). An Elastic Tube Theory of Pulse Transmission and Oscillatory Flow in Mammalian Arteries. Wright Air Development Center Technical Report. TR 56-6114, Dayton, OH).

