# Corrigendum: Estimation of left ventricular end-systolic elastance from brachial pressure waveform via deep learning

**DOI:** 10.3389/fbioe.2023.1341852

**Published:** 2023-12-05

**Authors:** Vasiliki Bikia, Marija Lazaroska, Deborah Scherrer Ma, Méline Zhao, Georgios Rovas, Stamatia Pagoulatou, Nikolaos Stergiopulos

**Affiliations:** Laboratory of Hemodynamics and Cardiovascular Technology, Institute of Bioengineering, Swiss Federal Institute of Technology, Lausanne, Switzerland

**Keywords:** cardiac monitoring, convolution neural networks, cardiovascular modelling, non-invasive, contractility

In the published article, there was an error. Wrong numbers have been used for the data train/validation/test split.

A correction has been made to the section **Material and Methods**, *Data Pre-processing*, Paragraph 1. This sentence previously stated:

“The train/validation/test split was set to be 60% (2,290 cases)/20% (764 cases)/20% (764 cases). By computing the MSE with decreasing training size, we noticed that similar results can also be achieved with fewer samples (e.g., 1,603) and, therefore, we may deduce that a training size of 2,290 is sufficient.”

The corrected sentence appears below:

“The train/validation/test split was set to be 60% (2,248 cases)/20% (750 cases)/20% (750 cases). By computing the MSE with decreasing training size, we noticed that similar results can be achieved with fewer samples (e.g., 1,603) and, therefore, we may deduce that a training size of 2,248 is sufficient.”

The authors apologize for this error and state that this does not change the scientific conclusions of the article in any way. The original article has been updated.

